# Allergy and related clinical symptoms among medical students and interns

**DOI:** 10.12669/pjms.35.4.1281

**Published:** 2019

**Authors:** Nahla Khamis Ibrahim, Abeer Ali Alghamdi, Mayar Majed Almehmadi, Asma Abdulwahed Alzahrani, Asraa Khairallah Turkistani, Khalid Alghamdi

**Affiliations:** 1 Nahla Khamis Ibrahim, Professor at Community Medicine Department, King Abdulaziz University, Jeddah, Saudi Arabia; Professor at Epidemiology Department, High Institute of Public Health, Alexandria University, Alexandria, Egypt; 2 Abeer Ali Alghamdi, Medical Intern, King Abdulaziz University, Jeddah, Saudi Arabia; 3 Mayar Majed Almehmadi, Medical Intern, King Abdulaziz University, Jeddah, Saudi Arabia; 4 Asma Abdulwahed Alzahrani, Medical Intern, King Abdulaziz University, Jeddah, Saudi Arabia; 5 Asraa Khairallah Turkistani, Demonstrator of Family and Community Medicine, Umm Al Qura University, Mecca, Saudi Arabia; 6 Khalid Alghamdi, Resident, King Abdulaziz University, Jeddah, Saudi Arabia

**Keywords:** Allergy, Clinical presentations, Predictors, Prevalence, Triggers

## Abstract

**Objectives:**

To determine the prevalence, types, clinical presentations, triggers, and predictors of allergic disorders among medical students and interns at King Abdulaziz University (KAU), Jeddah, Saudi Arabia.

**Methods:**

A cross-sectional design was used for this study in which 600 medical students and interns were selected by a multistage stratified random sampling. A validated, confidential, self-administered questionnaire was used during 2016 / 2017. It asked about the previous diagnosis of allergic disorders, associated factors, types, clinical symptoms and the triggering allergenic. Descriptive & inferential statistics were done and logistic regression analysis was conducted.

**Results:**

The overall prevalence of diagnosed allergic disorder(s) among the participants was 36.2%.The commonest types of allergy were skin (33.8%) followed by respiratory (29.5%) presentations. The most frequently reported allergenic triggers were the house dust (45.6%) and smoke (30.4%). The first allergy predictor was family history of allergic disorders (aOR= 4.35, 95 % CI: 2.96-6.39), followed by female gender. Regarding the outcome of allergy on students’ life, 16.1% occasionally missed classes, and 28.6% had sleep disturbance during allergic attacks.

**Conclusion:**

Allergy represents an important problem among medical students and interns. Family history and female gender were the predictors of allergy. Skin and respiratory allergies were the most common types. House dust and smoke were the commonest allergenic triggers. Detection of allergens and management of cases of allergy among medical students and interns are needed. Education and conduction of awareness campaigns about allergy are needed.

## INTRODUCTION

Allergy is a widespread global public health problem.^1^ It has continuously escalating rates, and it can lead to serious complications.^1^,^2^ The etiology of allergy is due to interactions between both environmental factors, with the permissive genetic factors.^3^ Allergy is a hypersensitivity reaction in which the immune mechanism responds (by IgE antibody) to the environmental materials (allergen) that are normally considered harmless.^4^

Allergic disorders have multi-organ presentations such as allergic rhinitis, asthma, urticaria, eczema and anaphylaxis.^1^ They are eco-system related conditions, with variations in predictors, and triggering allergens from different countries.^3^ The increasing rates of allergy may be attributed to changes in the lifestyles and living conditions.^2^ Such increase is well recognized globally during the previous few decades.^2^,^5^,^6^ Worldwide, allergic disorders affected up to one-third of the world’s populations.^7^ It was estimated, 2014, that 400 million persons had allergic rhinitis, 300 million suffered from asthma, and about 200-250 million complained from food allergies. Furthermore, it was estimated that 10% of the world’s people had allergy caused by drugs. Allergy can be associated also with increased economic burden, and results in an annual cost of billions of dollars.^8^ Urbanization and globalization resulted in marked environmental changes, and lead to increased allergens in the environment.^9^

Asthma was ranked as one of the most common chronic diseases in Saudi Arabia.^10^ During college years, allergies may affect students’ quality of life by interrupting their daily activities, diminishing faculty attendance, precipitating poor sleep quality, and diminishing the ability to accomplish academic and extra-curricular activities.^9^ A study conducted among 112 medical students from Albaha, Saudi Arabia, revealed a high prevalence of asthma symptoms.^11^

Nowadays, there is an increasing interest in the field of allergy among medical students.^12^ However, there is lack of adequate number of comprehensive epidemiological research done among a large sample of medical students from Jeddah. So, such study is required.The current study was done to determine the prevalence, types, triggers, and predictors of allergic disorders among medical students and interns from King Abdulaziz University (KAU), Jeddah.

## METHODS

A cross-sectional study was conducted between medical student (2^nd^ -6^th^ year), and interns at KAU during the academic year 2016 / 2017. A multistage stratified random sampling method was used. Stratification took into consideration the gender and the educational level. The sample size was calculated using the formula for estimation of sample size from a cross-sectional study.The prevalence ‘p” = 0.5, as the most conservative sample due to lack of similar studies from Jeddah. So, q=1−p= 0.5 & d was put at 0.04%. The minimum calculated sample was 600.

A validated, anonymous, confidential self-administered questionnaire was used. Face and content validity was done by two experts. Alpha Cronbach’s test was done for assessing the internal consistency reliability and was found to be 0.81.

Information about personal, socio-economic data, habits and chronic diseases were collected. Stress reported during the six months preceding the study, and family history of allergy were also asked. Data about the surrounding environment was assessed. The questionnaire inquired about participant’s allergy (≥1) according to previous physician’s diagnosis. If allergy was present, the type (skin, respiratory, eye, food, and anaphylaxis) was determined. For each type, the specific clinical presentations were also assessed. The participants were also asked about conduction of skin test and its result. They were also inquired about the type of received treatment (if any). Furthermore, the effect of allergy on the students’ life was assessed.

### Statistical analysis

Data analysis was done using the SPSS version 20. Descriptive and inferential statistics were done. A stepwise multiple logistic regression analysis was done to delineate the predictors of allergic disorders. *P-value <* 0.05 was considered statistically significant.

### Ethical consideration

The study was approved by the Institutional Review Board (IRB). The study followed the “ethical values of Helsinki declaration”. Official approvals were obtained. Each participant wrote an informed written consent.

## RESULTS

The mean age of participants was 22±1.7 years, with a male to female ratio of 1:1.05. Prevalence of diagnosed allergic disorders (≥ 1 type) among the participants was 36.2% as shown in Table-I. Cutaneous (33.8%), respiratory (29.5%) and eye allergies (11.2%) were the most prevalent types. The most frequently reported skin allergy was eczema (14.3%). Concerning respiratory allergy, the most prevalent types were nasal congestion & allergic rhinitis (AR). Asthma was reported by 6.8% of the participants. The commonest triggering factors of allergy were the house dust (45.6 %), smoke (30.4%) and cold air (28.6%). Table-II.

**Table I:** Prevalence, clinical types, presentations of the diagnosed allergic disorders among medical students and interns, King Abdulaziz University (n=600).

Type of allergy	No.	%
Any type of allergy (≥ 1)	217	36.2
Skin (cutaneous) allergy	203	33.8
Eczema (atopic eczema)	86	14.3
Contact dermatitis	54	9.0
Itchy rashes	41	6.8
General itching	36	6.0
Hives	22	3.7
Other symptoms (swelling, pimply rashes, etc.)	35	4.8
Respiratory or airway allergy (upper, lower)	177	29.5
Nasal congestion	106	17.7
Allergic rhinitis (AR)	100	16.7
Running nose	83	13.8
Asthma	41	6.8
Wheezy chest	37	6.2
Chest tightness	31	5.2
Shortness of breath	23	3.8
Hay fever	1	0.2
Others (post nasal drips, itchy nose, sinus allergy)	102	17
Ophthalmic allergy	67	11.2
Food allergy	64	10.7
Anaphylaxis	24	4.0
Food	10	1.7
Drug (e.g. penicillin)	8	1.3
Latex (rubber)	1	0.2
Other	5	0.8

**N.B.** Each question was separately asked

**Table II:** Frequencies of different triggering allergens among medical students & interns with allergic disorders at King Abdulaziz University (n=217).

Triggering allergens	No	%
House dust	99	45.6
Smoke	66	30.4
Cold air	62	28.6
Perfumes	58	26.7
Weather changes	58	26.7
Pollution	51	23.5
Cats	45	20.7
Odours	42	19.4
Grass	29	13.4
Formalin	28	12.9
Stress	27	12.4
Insecticides	26	11.9
Hay	21	9.7
Pets (dogs)	21	9.7
Humidity	21	9.7
Cosmetics	19	8.8
Mould & mildew	19	8.8
Exercise	17	6.5
Leaves	14	6.4
Basement floors	13	5.9
Latex	10	4.6
Milk/dairy products	9	4.1
Menstruation (females with allergy.= 134)	5	3.7

**NB:** Each question was separately asked

Females had much higher prevalence of allergy (43.5%) compared to males (28.4%).Table-III. A highly statistical significant difference was present (*X^2^*=14.8, *P*<0.001). Participants from families with higher income, and whose fathers obtained a university degree or above reported a higher rate of allergy than others. Smokers had a slightly higher prevalence of allergy compared to others (*P* > 0.05). Furthermore, allergy rate was slightly higher among those exposed to stress, during 6 months preceded the study, compared to others (*P* > 0.05). Participants with family history of allergic disorders were about 5 times more prone to have allergy compared to others (OR= 4.7, 95% C.I.: 3.22-6.87). In addition, those who complained of water leaks and molds contamination near their houses had a significantly higher prevalence of allergy than others (*X^2^*=3.98, *P* < 0.05).

**Table III:** Relationship between presence of allergy and the study variables among medical students and interns at King Abdulaziz University.

Variable	Allergy (n=217)		No allergy (n=383)		X^2^	(p)	OR (CI)

	No.	%	No.	%			
*Gender*							
Female	134	43.5	174	56.5	14.8	(0.000)	1.93 (1.38-2.72)
Male	83	28.5	209	71.6			
*Marital status*							
Single	202	35.9	361	64.1	0.3	(0.57)	0.82 (0.42-1.62)
Married	15	40.5	22	59.5			
*Educational level:*							
Basic	59	35.8	106	64.2	0.02	(0.90)	0.98 (0.67-1.42)
Clinical & interns	158	36.3	277	63.7			
*Father education*							
University or above	175	38.3	282	61.7	3.76	0.049	1.62 (1.07-2.46)
Less than university	42	29.4	101	70.6			
*Mother education*							
University or above	134	35.6	242	64.4	0.04	(0.84)	1.04 (0.73-1.47)
Less than university	78	34.8	146	65.2			
*Father Occupation*							
Professional	159	38.8	273	63.2	0.27	(0.6)	1.11 (0.78-1.65)
Non-professional	58	34.5	110	65.5			
*Mother occupation*							
Professional	96	36.8	165	63.2	0.07	(0.78)	1.05 (0.75-1.47)
Non-professional	121	35.7	222	64.3			
*Father income*							
Not enough, Enough	213	36.5	370	63.5	1.21^a^	(0.27)	1.87 (0.60-5.81)
Enough and exceed	4	23.5	13	76.5	(Fisher’s exact test)		
*Current smoker*							
Yes	29	39.2	45	60.8	0.34	(0.56)	1.16 (0.70-1.91)
No	188	35.7	338	64.3			
*Passive smoker*							
Yes	113	36.0	201	64.0	0.12	(0.73)	1.06 (0.76-1.49)
No	99	34.6	187	65.4			
*Stress*							
Yes	29	39.2	45	60.8	3.3	(0.07)	1.41 (0.97-2.05)
No	188	35.7	338	64.3			
*Family history of allergy*							
Yes	169	50.8	164	49.2	68.9	(0.000)	4.70 (3.22-6.87)
No	48	18.0	219	82.0			
*Wood /Coal Stove*							
Yes	43	35.8	77	64.2	0.02	(0.90)	1.03 (0.68-1.56)
No	169	35.2	311	64.8			
*Water leaks & molds contamination*							
Yes	25	49.1	26	50.9	3.98	(0.04)	1.79 (1.01-3.182)
No	192	34.9	357	65.1			

a Fisher’s exact test

First predictor of allergy among medical students and interns was having family history of allergic disorders (aOR= 4.35, 95% CI: 2.96-6.39), followed by being a female (aOR= 1.50, 95 % CI: 1.04-2.15) Table-IV.. Regarding medications, 62.2% of the participants who had allergy reported receiving drugs. The commonest received treatment were the antihistaminic drugs (30.4%), corticosteroids (11.1%), bronchodilators (10.4%) and decongestants (8.1%). In addition, 40.0 % of them used more than one treatment modalities (anti-histaminic, cortisone, decongestants, immunotherapy, etc). Those who used cortisone in any form (either separate or in combinations) accounted for 31.9%. Regarding the source of medications, 41.5% of the students with allergy used over the counter (OTC) drugs. In addition, 3.0% of them attended hospital for receiving oxygen, and 6.2% had never admitted to hospital for allergy. Regarding outcome of allergy, 16.1% of students with allergy reported missing their classes due to it, and 28.6% complained from sleep disturbance during allergic attacks.

**Table IV:** Predictors of allergic disorders among medical students and interns at King Abdulaziz University.

Variable	B	P	aOR	95% C.I.
Family history of allergy	1.471	0.000	4.35	2.96- 6.39
Gender (Female)	0.402	0.030	1.50	1.04 – 2.15
Constant	-1.656			

Among the participants diagnosed with allergy, only 30 (13.8%) conducted skin-prick test. Results found that the most common separate specific IgE (sIgE) allergens was related to foods as shrimps (8 cases; 26.7%), insects (4 cases; 13.3%), pet dander (4 cases; 13.3%), dust mites (3 cases; 10.0%) and moulds (3 cases; 10.0%). Furthermore, in 26.7% of the tested cases had a poly-sensitization to more than one allergens.

## DISCUSSION

The overall prevalence of allergy among our participants was 36.2%, which agrees with results of nine cohorts among third year medical students from Zurich, Switzerland,^1^ and with results among Turkish adolescents and adults.^6^ However, our rate is higher than rates reported between Polish female university students (2009 & 2015).^2^ This discrepancy may be due to differences between countries, populations, or time of studies. Increase environmental allergens of indoor and outdoor pollution &decreased biodiversity may contribute to the high allergy prevalence nowadays.^2^

Prevalence of allergy among our females is more than males, which agrees with results from Ajman, UAE.^9^ Estrogens endogenous female sex hormone may support allergic reactivity by working through estrogen receptor- α of mast cells.^13^ Our participants from families with better income and paternal education had higher prevalence of allergy than others. This may be because prevalence of IgE sensitization to aeroallergens is increased among persons with high salaries.^3^

Our study found that family history of allergy was the first predictor of allergy among participants, which agrees with other studies.^9^,^14^ This could be one evidence of the inheritance in allergy. These findings agree with the possible genetic effect in of allergy among family members^9^ and with the recent genetic researches which identified many genetic loci at different Interleukin (IL) “IL1RL1IL18R1, HLA-DQ, IL33, SMAD3, ORMDL3 GSDMB & IL2RB” and these are found to be related to allergic disorders.^15^

The skin is one of the largest immunologic organs that is affected by both external and internal factors, as well as by innate and adaptive immune responses.^16^ Our study also revealed that cutaneous allergy was the commonest type allergic disorders. This result coincides with that reported among medical students from Turkey.^17^ Regarding different clinical presentation of allergy, the current study showed that prevalence of eczema (atopic eczema) was 14.3%. Similar rates were reported from Ajman^9^, and Lebanon.^18^

Regarding respiratory allergy, the prevalence of AR from the current study was 16.7%, which coincides with results from a recent study, 2017, done among adolescents from Korea.^19^ On the other hand, our rate is higher than the rate of AR (10%) from household surveys in five Middle East countries; based on physicians’ diagnosis.^20^ The cause of such discrepancy may be due to differences between the age of the target populations, or the method of reporting and diagnosis the cases. Similarly, a lower rate of AR (7.0%) than that of our study was reported from seven Emirates of the UAE.^21^

The prevalence of asthma & wheezy chest among the participants from the present study were found to be *6.8%* and 6.2%, respectively. Our rate of asthma coincides with a rate (7.5%) from South India.^4^ However, a lower rate of asthma (4.05 %) was reported from a Saudi household survey done among population aged ≥15 years.^22^ The cause of this discrepancy may be due to differences between the age group and the types of the target populations.

Food allergy occurred among about one-tenth of medical students and interns in the current study. However, a higher rate (17%) was reported from Albaha university^11^, and this difference may be due to differences between the target populations. Concerning ophthalmic allergy,11.2% of our participants complained of it, which is much lower than the rate reported from Ajman.^9^ This may be due to the differences between both countries.

In the present study, drug-induced anaphylaxis (especially that induced by antibiotics) was reported by 1.3% of the participants. The prevalence of drug hypersensitivity reported among medical students from Turkey was 4.7%.^17^ The cause of lower rate from the current study may be because we inquired about anaphylaxis not all types of hypersensitivity. Regarding allergenic triggers, house dust was the most common trigger in the present study, which agrees with the results from Albaha^11^, Ajman^9^, South India^4^ and from the seven Emirates of UAE.^21^ House dust mite was one of commonest reported allergens between Swiss medical students.^1^ Urbanization have altered the housing conditions due to soft furnishings, close-fitting carpets, central air-conditions, and decreased indoor ventilation. Great increase in indoor pollution and allergens can lead to elevation of rates of allergy.^9^

Among our participants, smoke (30.4%) and perfumes (26.7%) were among the most frequently reported allergy triggers (following house dust). Similarly, a study from France, 2017^23^, concluded that the same allergens triggers were among the commonest asthmatic triggers.

Stress was reported as triggering factor of allergy in the present study, and the stressed students reported a higher rate of allergy compared to others. Stress can trigger allergy by causing inflammation via modulating immune function by both neural and hormonal mechanisms.^23^ Our results found that the commonest sIgE positivity was for foods as shrimps, which concurs with a study from China, 2017.^24^

The effect of allergies on life, and the extent to which it may restrict the daily activities are often ignored.^9^ In the present study, 16.1% of the participants who complained of allergy reported that they missed classes, and 28.6% of them complained of sleep disturbance during allergic attacks. The study of Ajman^9^ also reported that allergy lead to interference with students’ academic activities and with the social and extracurricular activities.

## CONCLUSION

The prevalence of the diagnosed allergic disorders was high (36.2%) among medical students and interns at KAU. The predictors of allergic disorders were family history of allergy and the female gender. Cutaneous and respiratory were the commonest types of allergies. The most important reported allergenic triggers were house dust, smoke, cold air, and perfumes. Regarding the outcome of allergy, 16.1% of students with allergy reported missing their classes due to it, and 28.6% complained from sleep disturbance; during allergy attacks. Detection of allergens is needed for avoiding them. Conduction of awareness campaigns about allergy, triggers and preventive measures are also needed. Screening and management of allergy cases is needed.

### Authors’ Contribution

**NKI:** Selected the topic, designed the study, analyzed data, writing, editing manuscript & the corresponding author.

**AAA, MMA, AAA, AKT& KA:** Data collection & entry, helped in statistical analysis and in manuscript writing.
